# Phytochemical Content and Pharma-Nutrition Study on* Eleutherococcus senticosus* Fruits Intractum

**DOI:** 10.1155/2016/9270691

**Published:** 2016-10-24

**Authors:** Daniel Załuski, Marta Olech, Agnieszka Galanty, Robert Verpoorte, Rafał Kuźniewski, Renata Nowak, Anna Bogucka-Kocka

**Affiliations:** ^1^Department of Pharmacognosy, Ludwik Rydygier Collegium Medicum, Nicolaus Copernicus University, 9 Marie Curie-Skłodowska Street, 85-094 Bydgoszcz, Poland; ^2^Department of Pharmaceutical Botany, Medical University of Lublin, 1 Chodźki Street, 20-093 Lublin, Poland; ^3^Department of Pharmacognosy, Collegium Medicum, Jagiellonian University, 9 Medyczna Street, 30-688 Cracow, Poland; ^4^Natural Products Laboratory, Institute of Biology, Leiden University, 2300 RA Leiden, Netherlands; ^5^Department of Biology and Genetics, Medical University of Lublin, 4a Chodźki Street, 20-093 Lublin, Poland

## Abstract

In the past two decades public interest in herbal products has increased significantly in Europe, especially in the plant-based products from non-European traditions.* Eleutherococcus senticosus* has been used for the treatment of inflammatory diseases, anemia, and rheumatoid arthritis. The* Eleutherococcus senticosus* fruits intractum was examined for the content of phenolic acids (LC-ESI-MS/MS), minerals (AAS), TPC, and TFC (spectrophotometric assay). The antioxidant activity was determined using free radical scavenging assay and TLC-DB-DPPH^∗^ dot-blot test. An anti-Hyal activity was evaluated by the spectrophotometric assay method. Cytotoxicity towards HL-60, HL-60/MX1, HL-60/MX2, CEM/C1, and CCRF/CEM leukemic cell lines was done using trypan blue test. Among eight phenolic acids,* trans*-caffeic acid was found in the largest amount (41.2 mg/g DE). The intractum presented a high amount of macroelements (Ca, Mg, K; 1750, 1300, and 21000 mg/kg) and microelements (Fe, Mn; 32.7, 54.3 mg/kg), respectively. The content of TPC and TFC was 130 and 92 mg/g DE, respectively. The intractum showed anti-Hyal activity (2.16–60%) and an antioxidant capacity (EC_50_; 52 *μ*g/mL). The intractum most strongly inhibited the growth of HL-60, HL-60/MX1, and CCRF/CEM. A better understanding of the intractum health benefits is important in order to increase its utility and enrich dietary sources of health promoting compounds.

## 1. Introduction

 Plant-based products have been used to manage various ailments for the centuries in different ethnic communities of the world. Nowadays, such products are used in developed and developing countries separately and/or together with synthetic drugs. It is established that about 120 plant derived compounds are used in western medicine, and about 80% of the world population uses medicinal plants in primary health care [1].

*Eleutherococcus senticosus* (Rupr. et Maxim.) Harms, called Siberian ginseng, is a medicinal plant with a long history of use (by the Chinese for over 2000 years). The plant has been recognized as an adaptogen, similarly to* Panax ginseng* [C. A. Meyer. (Araliaceae)],* Schisandra chinensis* [Turcz. Baill (Schisandraceae)], or* Aralia mandshurica* [Rupr. et Maxim. (Araliaceae)].

In the Chinese and Russian ethnomedicine, its use was empirical, because people used to believe that it was a panacea that promoted longevity, with beneficial effects for the treatment of physical fatigues. The fruits have been used for a long time as an ingredient of the fermented wine, the leaves as a tonic, as a functional beverage marketed for reducing liver damage, and accelerating alcohol detoxification [2–5].

At present, in China the ethanol extract of the roots is a popular health supplement for weakness, diseases connected with inflammation (rheumatism, haemorrhoids), and impotence. It was reported that, in the Olympic Games, the players of the Old Soviet Union have increased records after administering the* E. senticosus* roots products [6, 7]. According to Załuski's previous studies, the fruits of that species cultivated in Poland, act as antioxidants, inductors of the apoptosis in Jurkat 45 and HL60 leukemic cell lines, and inhibitors of MMP-1, MMP-2, MMP-3, and MMP-9 [8, 9].

The* Eleutherococcus senticosus* products attract global attention as a novel medicinal plant and since a few years have become popular as dietary supplement in the United States and European countries. Imported products of this plant have become available in North America, with a market share of 3.1% of the $12 billion medicinal herbal industry. The 1994 DSHEA (Dietary Supplement Health and Education Act) regulation allows a direct commercialization of* E. senticosus* as a supplement for consumption in the United States without the regulation of the FDA (Food and Drug Administration). With the increased awareness of developing countries that the study of traditional medicines and finding new leads is important, there is a need to avoid imported expensive eastern medicines and to estimate a new source of some eastern herbs in Europe. Preparations of the roots of* E. senticosus* are given in cases of asthenia with weakness and fatigue, for example, in convalescence. This indication has been officially accepted by the Community Herbal Monograph on* Eleutherococcus senticosus* (Rupr et Maxim) Maxim Radix (EMEA/HMPC/244569/2006), published by the European Medicines Agency. The clinical application of* E. senticosus* is generally considered safe; however, the European Community Herbal Monograph states “arterial hypertension” as a contraindication [5–7].

**Figure 1:**
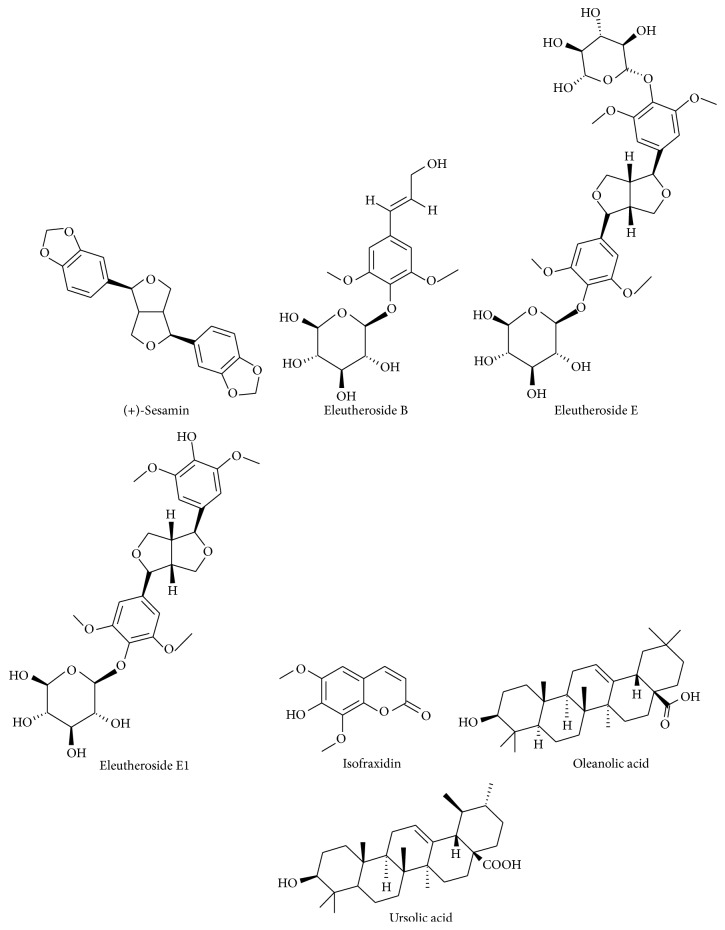
The chemical structures of main* E. senticosus* compounds [9].

The roots of* E. senticosus* are source of phenols, called eleutherosides (derivatives of lignans, coumarins, and phenylpropanoids), flavonoids (hyperin, rutin, afzelin, quercetin, and kaempferol), phenolic acids, triterpenic acids, and anthocyanins (Figure 1). Compounds isolated from the fruits belong to eleutherosides (eleutherosides B and E), flavonoids, phenolic acids, and essential oil (0.3%, v/d.w.). The dried fruits, consumed as food, are rich in Ca, Mg, Mn, Zn, and Cu. In the leaves, flavonoids (quercetin, quercitrin, and rutin) have been identified [8–11].

*Eleutherococcus senticosus* is an example of one such species, whose activity and chemistry are yet to be studied in more detail, especially the species harvested in different places. The* Eleutherococcus senticosus* products, which are available in the herbal drugs market, are imported from China. Because of a lack of the assessment of plant material there have been many cases of the poor quality of plants supplied by Chinese traders, leading to financial losses for some pharmaceutical companies. For example, 26% of products prepared, among others, from* E. senticosus* did not meet label claims with respect to the claimed eleutherosides content [12]. To avoid that, the establishment of the new source of important medicinal plants in Europe is required. As a major part of western medicine has been developed from traditional knowledge, it makes sense to first of all look to our ancestors' knowledge and study that with all the novel concepts and source of the plant material.

The chemical compounds and biological activity of plants depend on the geographical zone of the growth. This species is successfully cultivated at the botanical garden in Rogów, which lies in the Central Polish Lowlands region with geographic data such as 51° 49′N and 19° 53′E. The average, long-term temperature is −20.1°C, what classified the garden to the 6bth subclimate (according to USDA Frost Hardiness Zones) and to the second zone according to the Kórnik's category. These plants are grown on the acidic, luvic, and sandy soils [13].

In view of several ethnopharmacological, medicinal, and nutritional value of the fruits described in literature and on the basis of Załuski's previous studies, it was decided to examine the fruits intractum for its phytochemicals and bioactivity. Many products, which are widely available in the health food or herbal drugs markets, are in the form of capsule, powder, or tea bag. So far the intractum is not available on market. As part of a program to search for bioactive constituents from* Eleutherococcus* species, this study was focused on the establishment of phenolic compounds (phenolic acids, TPC, and TFC), minerals content, and anti-Hyal, anti-DPPH^*∗*^, and cytotoxic properties of the intractum prepared from the fruits harvested in Poland.

## 2. Materials and Methods

### 2.1. Standards and Reagents

Folin-Ciocalteu reagent, DPPH, ascorbic acid, DMSO, bovine albumin, hyaluronidase from bovine testes type I-S,* Streptococcus equi* hyaluronic acid, and sodium phosphate buffer pH 7.0 were obtained from Sigma-Aldrich. DNPH and FeCl_3_ and ethanol were obtained from POCH (Lublin, Poland). The acetate buffer, pH 4.5, was purchased from J. T. Baker, USA. The standards of phenolic acids, flavonoids, and aescin were obtained from ChromaDex (Santa Ana, CA). Liquid chromatography- (LC-) grade methanol (MeOH) and acetonitrile (ACN) were purchased from Merck (Darmstadt, Germany). LC-grade water was prepared using a Milli-Q purification system (Millipore, Bedford, MA, USA). All others reagents were of analytical grade.

### 2.2. Plant Material

The fruits of* E. senticosus* (Rupr. et Maxim.) Maxim. were collected at the arboretum in Rogów (Poland) in October 2015 (voucher specimen number ES/09/14F). The sample was deposited at the Department of Pharmacognosy, Collegium Medicum, Bydgoszcz, Poland. Immediately after harvesting, the intractum was prepared.

### 2.3. Preparation of Intractum

20 g of fresh fruits was macerated in 100 mL 40% ethanol for 30 days at room temperature. After that, the extract was filtered through Whatman number 4 filter paper. The solvent was dried with an evaporator under vacuum conditions at 45°C and the residue was subjected to lyophilisation. The extraction yield was calculated based on the dry weight of the extract and was expressed as a percentage calculated according to the formula:

(1) $$\begin{array}{c} \%\text{ Yield}=\frac{\text{extract weight}}{\text{sample weight}}\times 100\%. \end{array}$$

### 2.4. Acidic Hydrolysis of Intractum as Sample Preparation before in LC/MS Analysis

1 g of intractum was hydrolysed in 1 mL of H_2_SO_4_ in 20 mL of 20% aqueous methanol. After refluxing at 80°C for 2 h, the extract was allowed to cool and dissolved in 20 mL of dichloromethane (left for 24 h, room temperature). Next, the liquid-liquid extraction was performed using 5 × 20 mL of ethyl acetate. Dichloromethane and ethyl acetate layers were evaporated, and the residues were used in LC/MS analysis.

### 2.5. Total Phenolic Content (TPC)

The total phenolic content of extracts was determined using the method of Singleton and Rossi [14]. Gallic acid was used to calculate the calibration curve (20–100 *μ*g/mL; *y* = 0.0026*x* + 0.044; *r* ^2^ = 0.999), and TPC was expressed as gallic acid equivalents (GAE/mL) as well as per typical administered dose (*i.e.,* per 25 mL intractum dose). The experiments were done in triplicate.

### 2.6. Total Flavonoid Content (TFC)

The TFC in investigated samples was determined using FeCl_3_ and DNPH colorimetric methods [15]. TFC were expressed as means (±SE) mg of quercetin equivalent (QEs/mL for FeCl_3_ method; 20–100 *μ*g/mL; *y* = 0.0041*x* + 0.236; *r* ^2^ = 0.999) and as means (±SE) mg of hesperetin equivalent (HEs/mL for DNPH method; 250–1000 *μ*g/mL; *y* = 6.374*x* − 0.098; *r* ^2^ = 0.988) as well as per typical administered dose (i.e., per 25 mL intractum dose). The experiments were done in triplicate.

### 2.7. LC-ESI-MS/MS Conditions of Analysis of Phenolic Acids

The samples were analyzed according to modified method of Nowacka et al. [16]. For this purpose an Agilent 1200 Series HPLC system (Agilent Technologies, USA) equipped with a binary gradient solvent pump, a degasser, an autosampler, and column oven connected to a 3200 QTRAP Mass Spectrometer (AB Sciex, USA) equipped with an electrospray ionisation source (ESI) and a triple quadrupole-ion trap mass analyzer was used. The separation of the analytes was carried out on a Zorbax SB-C18 column (2.1 × 50 mm, 1.8 *μ*m particle size; Agilent Technologies, USA) maintained at 25°C, using 3 *μ*L injections. The solvents used were water containing 0.1% HCOOH (solvent A) and methanol containing 0.1% HCOOH (solvent B). The following gradient elution program at a flow rate of 370 *μ*L min^−1^ was applied: 0-1 min, 5% B; 2–4 min, 20% B; 8–9.5 min, 70% B; 11.5–15.5 min, 5% B. Mass spectrometer was controlled by the Analyst 1.5 software. ESI worked in the negative-ion mode with the curtain, nebulizer, and turbo-gas (all nitrogen) set at 30, 60, and 60 psi, respectively. The ion spray needle voltage was −4500 V and capillary temperature 400°C. For each compound the optimum conditions of Multiple Reaction Mode (MRM) were determined in the direct infusion mode. Triplicate injections were made for each standard solution and sample. The analytes were identified by comparing retention time and *m*/*z* values obtained by MS and MS^2^ with the mass spectra from corresponding standards tested under the same conditions. The calibration curves obtained in MRM mode were used for quantification of all analytes. The identified phenolic acids were quantified on the basis of their peak areas and comparison with a calibration curve obtained with the corresponding standards. Linearity ranges for calibration curves were specified. The limits of detection (LOD) and quantification (LOQ) for phenolic compounds were determined at a signal-to-noise ratio of 3 : 1 and 10 : 1, respectively, by injecting a series of dilute solutions with known concentrations. Detailed LC-ESI-MS/MS methods parameters are given in Supplementary Material (see Supplementary Material available online at http://dx.doi.org/10.1155/2016/9270691).

### 2.8. AAS of Minerals

0.5 g of the lyophilised intractum was put into a burning cup, and 2 mL of pure HNO_3_ was added. The samples were incinerated in a MARS 5 microwave oven (Manufactured by, CEM corporation, USA) at a temperature of 90°C for 15 min, next 120°C for 10 min, and 210°C for 30 min, and the solution was diluted to 100 mL with water. Concentrations were determined with an Varian SpektrAA 280FS + Autosampler SPS 3 Spectrometer. Minerals and trace elements were determined using the instrumental conditions recommended for each mineral and were calculated based on the respective standard curve.

### 2.9. Antihyaluronidase Studies

The ability of the extracts to inhibit Hyal was determined by the spectrophotometric method of Yahaya et al. [17]. The intractum concentration was 1.0 mg/mL in 10% water ethanol solution. The final concentration in the reaction's mixture was 22 *μ*g/0.161 mL. The activity was determine on the basis of precipitation of undigested HA with albumin. 50 *μ*L of enzyme in acetate buffer pH 4.5, 50 *μ*L of sodium phosphate buffer (50 mM, pH 7.0; with 77 mM NaCl and 1 mg/mL of albumin), and 22 *μ*L of the analyzed samples were combined. All the reaction mixtures were incubated at 37°C for 10 min. Next, 50 *μ*L of HA (0.3 mg/mL of acetate buffer pH 4.5) was added and incubated at 37°C for 45 min. The undigested HA was precipitated with 1 mL acid albumin solution made up 0.1% bovine serum albumin in 24 mM sodium acetate and 85 mM acetic acid. The mixture was kept at room temperature for 10 min.; the absorbance of the reaction mixture was measured at 600 nm (Multidetection Microplate Reader SynergyTM HT, BioTek). Aescin was used as the positive control at the following concentrations: 0.05, 0.1, 0.2, 0.4, 0.6, and 0.8 mg/0.161 mL; the absorbance in the absence of enzyme was used as the blind control. All assays were done in triplicate. The percentage of inhibition was calculated as

(2) $$\begin{array}{c} \%\text{ inhibition}=\left[\;\frac{\left(AB-AE\right)}{\left(AS-AE\right)}\;\right]\times 100, \end{array}$$

where AB is absorbance of the enzyme + substrate + extract; AE is absorbance of the enzyme + substrate sample; AS is absorbance of the enzyme + substance sample.

### 2.10. Cytotoxic Activity

Leukemic cells HL-60-human Caucasian promyelocytic leukemia from American Type Culture Collection (ATCC CCL-240™), HL-60/MX1-human Caucasian acute promyelocytic leukemia from American Type Culture Collection (ATCC CRL-2258™), and HL60/MX2-human Caucasian acute promyelocytic leukemia from American Type Culture Collection (ATCC CRL-2257™), CEM/C1-human Caucasian acute lymphoblastic leukemia from American Type Culture Collection (ATCC CRL-2265™), and CCRF/CEM-human Caucasian acute lymphoblastic leukemia from American Type Culture Collection (ATCC CCL-119™) were incubated at the concentration of 5 × 10^5^ cells/mL in 5% CO_2_ atmosphere for 24 h at 37°C. An RPMI 1640 medium (Sigma, St. Luis, USA), with 15% fetal bovine serum (Sigma), 2 mM L-glutamine and antibiotics [100 U/mL penicillin, 100 *μ*M/mL streptomycin, and 2.5 *μ*g/mL amphotericin B (Gibco, Carlsbad, USA)] served as a growing medium.

The* in vitro* cytotoxicity assay was carried out using trypan blue assay. The cell lines, at concentration 5 × 10^5^ cells/mL, were treated with different concentrations of testing intractum and incubated for 24 h at 37°C in air atmosphere humidified by 5% CO_2_. At the end of this period, the medium was removed from each plate by aspiration. Next, the cells were washed with PBS and centrifuged at 800 rpm for 10 min, and then PBS was removed by aspiration. Then 10 *μ*L suspension cells were incubated for 5 min with the 10 *μ*L 0.4% trypan blue solution (Bio-Rad Laboratories, Inc., Hercules, CA). The samples were analyzed using an Olympus BX41 light microscope. The cells were stimulated with the ethanol extracts from the roots and leaves dissolved in DMSO at the final concentration from 10 to 100 *μ*g/mL of cell culture. The final concentration of DMSO in incubating mixture was 1%. The experiments were done in triplicate. As a positive control podophyllotoxin was used.

### 2.11. DPPH Assay

The ability to scavenge DPPH^*∗*^ free radicals was determined by the modified method of Brand-Williams et al. [18]. The methanol solutions of the extracts at the following concentrations, 0.1; 0.5; 1.0; 1.5 mg/mL, were used. Ascorbic acid and tocopherol were used as positive control (12.5; 25; 50; 75 *μ*g/mL). Absorbance was measured on a Multidetection Microplate Reader SynergyTM HT, BioTek. The experiments were done in triplicate. The sample concentrations providing 50% of scavenging (EC_50_) were calculated from the graph plotted between the percentages of scavenging and the sample concentrations.

### 2.12. TLC-DB-DPPH Dot-Blot Test (TLC-Direct Bioautography Dot-Blot Test)

The TLC-DPPH dot-blot test was used, 1 *μ*L of extracts (10 *μ*g/*μ*L) was applied on silica gel plates, and the plate was immersed for 5 s in freshly prepared 0.2 mmol methanolic DPPH^*∗*^ solution. After removing DPPH^*∗*^ excess, the decolourization of DPPH^*∗*^ was observed after 1, 5, 10, and 30 min.

### 2.13. Statistical Analysis

All determination was done in triplicate. The obtained data were subjected to statistical analysis using Statistica 7.0. (StatSoft, Cracow). The evaluations were analyzed for one-factor variance analysis. Statistical differences between the treatment groups were estimated by Spearman's (*R*) and Person's (*r*) test. All statistical tests were carried out at significance level of *α* = 0.05.

## 3. Results and Discussion

### 3.1. TPC and TFC

The literature regarding the pharmaceutical and dietary products of* Eleutherococcus senticosus* is scare, and this study may contribute to confirming the ethnopharmacological use of this plant. The extraction of the fruits resulted in 4.51% dry extract yield. After lyophilisation, the intractum was a dark red paste, with a characteristic smell and with a very sweet taste. Taking into consideration various preparations in liquid or solid dosage forms for oral use, we calculated the amount of TPC and TFC as mg/mL, mg/per serving, and mg/g exc. The results revealed a significantly high amount of TPC and TFC (Table 1). It is worth noting that the intractum contains more flavanones and dihydroflavonols (73.5 mg/g DE), the compounds whose occurrence in nature is limited, than flavone/flavonols (18.5 mg/g DE).

The TPC and TFC were higher than those previously reported for the various* Eleutherococcus* species, cultivated in Poland. Załuski et al. [8] reported on the TPC in the 75% ethanolic extracts from the spring leaves (20.3 to 37.2 mg/g), fresh fruits (6.1–19.7 mg/g), and roots (6.9–10.6 mg/g). In addition to this, the content of TPC and TFC in the fruits is not changed during a storage at room temperature. After 1-year storage the amount did not change significantly and was between 3.85 and 4.13 g/100 g for* E. senticosus* and* E. henryi*, compared to the freshly dried fruits (4.11 to 4.35 g/100 g), [19]. Heo et al. [20] studied the ethanol, methanol, and water extracts of the* E. senticosus* fruits growing in Korea but reported a lower concentration of polyphenols and flavonoids than that now estimated (0.3; 0.6; 0.6% and 0.20; 0.23; and 0.3%, resp.), which is also in agreement with the studies of Shohael et al. [21]. According to Jang et al. [22] the fruits of* E. senticosus* collected in Korea have contained the TPC and TFC in the range of 197.9–334.3 mg/g and 41.2–203.7 mg/g.

Our findings revealed that the intractum contains more the TPC than the blueberries or rose fruits, which in Poland or other European countries are very widely used in food products, for medical purposes, and are recognized as a rich source of polyphenols. According to Grace et al. [23] blueberries contained from 22.7 to 39.3 mg/g extract of polyphenols. Nowak et al. [24] found that tincture of the rosehip contains 11.8 mg/mL.

The high content of polyphenols and flavonoids may result from the polarity of ethanol that can penetrate the cellular membrane and dissolve the intracellular constituents in the plant cells. Another factor may be time of the intractum preparing, 30 days of storage.

### 3.2. LC-ESI-MS/MS of Phenolic Acids Content

**Figure 2:**
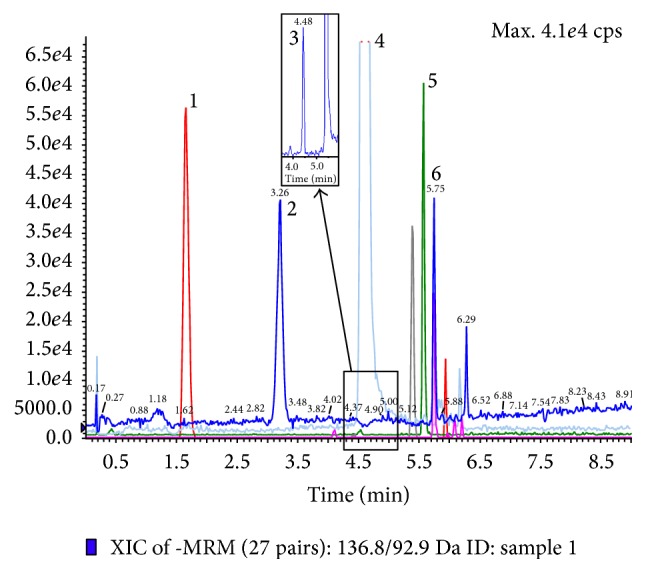
Chromatogram of phenolic acids (acquired in MRM mode) found in the intractum. Monitored MRM transitions are given in brackets: 1: protocatechuic acid (*m*/*z* 152.9→107.8); 2: 4-hydroxybenzoic acid (*m*/*z* 136.8→92.9); 3: vanillic acid (*m*/*z* 166.8→107.9), 4:* trans*-caffeic acid (*m*/*z* 178.7→134.9); 5:* trans*-p-coumaric acid (*m*/*z* 162.7→119), and 6:* trans*-ferulic acid (*m*/*z* 192.8→177.9). Unnumbered peaks represent uncharacterized constituents. Please refer to Supplementary Material for details.

The different types of compounds present in the intractum and the obtained chromatogram are presented in Figure 2. Compounds were identified by comparison of retention times and mass fragmentation pattern with data obtained for commercial standards and molecular masses were clearly recognized from the negative ESI-MS spectra. The obtained results are presented in Supplementary Material (Tables S1 and S2). Among eighteen phenolic acids (gallic, protocatechuic, gentisic, 4-OH-benzoic, 3-OH-benzoic, vanillic,* trans*-caffeic,* cis*-caffeic, syringic,* trans*-*p*-coumaric,* trans*-ferulic, veratric, salicylic, 3-OH-cinnamic,* trans*-sinapic, and* cis*-sinapic) just eight were found and quantitatively determined. The concentrations of individual compounds, which were quantified by comparison of peak areas with the calibration curves obtained for the corresponding standards, are reported in Table 2. To the best of our knowledge there are no studies investigating the profiles of phenolic acids in the intractum. The findings revealed the presence of benzoic and cinnamic acid derivatives; among them, a majority is present in a glycosidic form. The raw intractum was found to be rich in vanillic and* trans*-caffeic acids (4.2 and 41.2 mg/g DE, resp.). It also contained significant amount of* trans*-ferulic,* trans-p*-coumaric, and 4-OH-benzoic acid. Analysis of hydrolysates provided additional information about composition of fruit intractum. Vanillic acid was the most abundant one in dichloromethane and ethyl acetate layer. Moreover, large quantities of 4-OH-benzoic acid (7.0 and 7.4 mg/g DE, resp.) were found.

Data in the literature indicated that Kurkin et al. [25] identified free phenolic acids (syringic,* p*-coumaric, vanillic,* p*-hydroxybenzoic, caffeic, and ferulic acids) and depside (chlorogenic acid) in the roots of* E. senticosus* growing in Russia. In turn, Li et al. [26] identified protocatechuic, chlorogenic, and caffeic acids in the roots of Chinese sample. Bączek identified rosmarinic, chlorogenic, ferulic, and caffeic acids in the roots, fruits, and stem barks of six species [27, 28]. It is worth noting that the intractum contains* trans*-caffeic acid, whose activity is beneficial for health. That compound is present in other plant species used in traditional medicine or human diet. A content of* trans*-caffeic acid in* Allium cepa* L. was dependent on a type of raw material. The highest content was determined in a dried material (22.4 *μ*g/g) contrary to a fresh material (0.17 *μ*g/g) [29]. Caffeic acid occurred in smaller amount in rose petals (0.14 *μ*g/g/DE) [24].

### 3.3. Mineral Composition

The diet of the European population is mainly composed of starchy foods, fruits, vegetables, meat, eggs, and milk. Because starchy foods are present in a majority of meals, many people suffer from nutrient's deficiencies [30–32]. Therefore, there is nowadays a growing interest in products with high nutripharmacological value. For this reason, the minerals content of the intractum was evaluated. The concentrations [mg/kg of dry weight] of the mineral components of the intractum according to the mineralisation and identification methods are reported in Table 3. The intractum presented a high amount of macroelements (Ca, Mg, and K; 1750, 1300, and 21000 mg/kg, resp.) and microelements (Fe, Mn; 32.7, 54.3 mg/kg, resp.).

Heo et al. [20] reported on mineral composition of the* E. senticosus* fruits growing in Korea. That species contains 465; 1433; 199; and 13 mg/kg dry weight of Ca, Mg, Mn, and Zn, respectively. Compared with species cultivated in Poland there is a wide variation in the level of Ca and Mn. The Polish one contains higher level of Ca and Zn but lower level of Mg and Mn. A major finding is that the species native to Asia does not contain Fe, the factor that excludes these fruits as an ingredient of antianemic diet. It is worth noting that the investigated samples have higher Fe concentration (32.7 mg/kg) than the* Rosa canina* L. and* Rosa damascena* Mill. fruits (27.0 and 11.0 mg/kg, resp.), which are very popular in the diet of the Europeans. According to a WHO report, over 2000 million people in developing countries have iron deficiency anemia [33]. In that case, the intractum should be considered a new dietary ingredient that may be included in the diet. Moreover, the results obtained in the present study indicated higher content of Mn, Zn, and Cu than that in the* Rosa canina* L. and* Rosa damascena* Mill. fruits [34]. It should be mentioned that the intractum also contains more Zn and Mn than walnut of kernels (from 17.9 to 20.6 and 17.5 to 22.2 mg/kg).

### 3.4. Antihyaluronidase Activity

**Figure 3:**
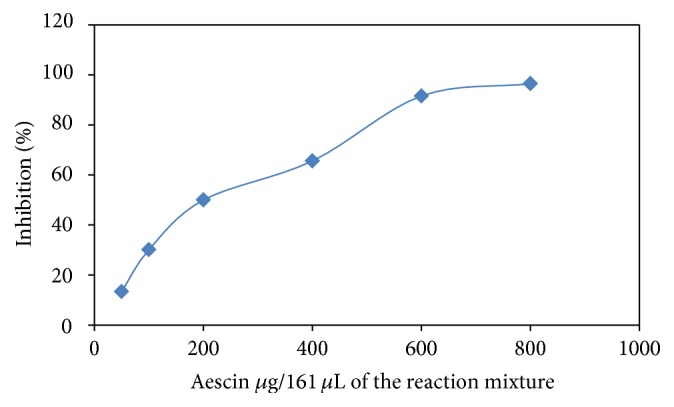
Inhibition of Hyal by aescin as the standard compounds (%).

Hyaluronidases belong to the hydrolases and digestive hyaluronic acid. Many investigators have reported a positive correlation between the expression of Hyal and the development of inflammation and tumor invasiveness. It was found that its activity is growing up in several diseases, such as the borderline tumors (108.9 mIU/mg protein), the benign epithelial tumors (89.2 mIU/mg protein), the functional cysts (53.4 mIU/mg protein), and malignant epithelial tumors (48.7 mIU/mg protein), in contrary to the normal ovaries (38.5 mIU/mg protein). Therefore, identification and characterization of Hyal inhibitors would be valuable for developing antitumor and anti-inflammatory agents. Recently, new hyaluronidase inhibitors are urgently required in the clinic, and there are none. In this study the impact of the intractum, at the inhibitor concentration 22 *μ*g/0.16 mL of the reaction mixture, was measured. In order to compare the antienzymatic activity of the intractum analyzed, aescin was used as the standard compound because of its well-recognized activity. Aescin inhibited Hyal at the level of 100% at concentration 800 *μ*g/0.16 mL of the reaction mixture (Figure 3).

As it is seen in Table 4, the intractum showed the highest inhibition in comparison to aescin and naringenin. The decrease in the inhibitory activity of compounds correlated with the increase of enzyme's unit. No inhibition was found in case 109 mIU. The high inhibition by intractum may result from the nature of chemical constituents of the intractum as a mixture. It is known that the activity of Hyal is dependent on the presence of Ca^2+^ [19]; in turn, the intractum contains a high amount of polyphenols acting as chelators.

Because there are no general procedures for anti-Hyal assay, it is hard to compare the results to others. The major factor is different enzyme's unit taken into assays, which excludes a reliable comparison. Therefore, in our work we decided to take the enzyme activity like in the above diseases. Despite a lack of a large amount of scientific literature, we tried to compare our results to the others. Bralley et al. [35] reported on anti-Hyal activity of the ethanol extracts of the* Vitis rotundifolia*. Michx peels and seeds of Early Fry (bronze) and Ison (purple)* Vitis rotundifolia* varieties. The obtained results indicated lower activity of the peel extracts contrary to the seed ones (IC_50_ 1.0, 1.0 for the Ison and Early Fry peels and 0.3, 0.6 for the Ison and Early Fry seeds; mg/mL). Considering isolates from the plant extracts, inhibitors of Hyal have been mainly found in flavonoids and triterpenes. Among flavonoids 7-O-butyl naringenin had a high value with 44.84% inhibition at 200 *μ*M concentration. Taking into account the chemical structure of flavonoids, their inhibitory activity towards Hyal increases with the number of hydroxyl groups, especially in 2,3′ position (quercetin) and 5′ (myricetin). Some authors state that the inhibition is dependent on the number of free, available hydroxyl groups and extension of side-chains. It was noticed that the inhibitory activity was decreased after glycosylation or substitution of hydroxyl groups. In turn, another compound, chlorogenic acid inhibited Hyal with IC_50_ 2.25 mM. A promising source of Hyal inhibitors may be some compounds present in essential oil, such as*β*-caryophyllene (IC_50_ 4.16 *μ*g/mL) and 1.8-cineole (IC_50_ 1.17 mg/mL). The mechanism of action may be related to the formation of complexes of polyphenols present in the extracts with ions present in the reaction medium [19, 36].

### 3.5. Analysis of Cell Viability

During the last few years an increase of leukemia evidences has been noticed. Leukemia is one of the most frequently occurring diseases among young people up to 30 years of age. Because of growing resistance to drugs, the treatment of leukemia is very difficult; therefore new drugs with antileukemic or strengthening body properties are still being searched for. In addition to this, it was confirmed that a higher activity of Hyal is in close correlation with metastasis and inflammation. Therefore, the inhibition of Hyal and the cytotoxicity of intractum was evaluated.

Five leukemic cell lines, HL-60, HL-60/MX1, HL-60/MX2, CEM/C1, and CCRF/CEM, were used. These cell lines represent different types of leukemia, namely, acute leukemia and myeloma. As a positive control podophyllotoxin was used; the results were published previously by Och et al. [37].

As presented in Table 5, the highest mortality of cells was observed in HL-60 cell line with the IC_50_ value of 10.4 *μ*g/mL.

Previous studies on cytotoxic activity of* E. senticosus* have shown a significant cytotoxicity towards HL-60 and Jurkat 45 leukemia cell lines. The ethanol extracts from roots and spring and autumn leaves revealed the IC_50_ values at concentrations 208, 312, and 299 *μ*g/mL towards HL-60. In turn, the chloroform extract from the roots (IC_50_ 2.8 *μ*g/mL), the ethanol extracts from the roots (134.7 *μ*g/mL), the spring leaves (199.7 *μ*g/mL), the fresh fruits (331.2 *μ*g/mL), and the dried fruits (274.4 *μ*g/mL) affected Jurkat 45 leukemia cell line [8, 38].

Varamini et al. [39] examined five quinoline alkaloids isolated from* Haplophyllum canaliculatum* Boiss. (7-isopen-tenyloxy-*γ*-fagarine, atanine, skimmianine, flindersine, and perfamine) against resistant (HL-60/MX1) and sensitive (HL-60) acute leukemia cell lines. 7-Isopen-tenyloxy-*γ*-fagarine appeared to have the highest IC_50_ value equal to 2.2 and 31.6 *μ*g/mL for HL-60/MX1 and HL-60, respectively. Tayarani-Najaran et al. [40] have investigated the cytotoxic effect of CH_2_Cl_2_ extract on HL-60 with the IC_50_ value of 68.83 *μ*g/mL while the intractum tested in our work exhibited 7-fold lower IC_50_ value (10.41 *μ*g/mL). Omosa et al. [41] tested 145 extracts from 91 Kenyan flora towards CCRF/CEM cell lines. The highest cytotoxic activity exhibited the ethanol extract of the berries of* Solanum aculeastrum* and 50% MeOH in CH_2_Cl_2_ extract of the stem bark of* Albizia schimperiana* with IC_50_ values of 1.36 and 2.97 *μ*g/mL. The other extracts that showed good activity included the extracts of the stem barks of* Zanthoxylum gilletii* (5% MeOH-H_2_O) and* Bridelia micrantha* 50% CH_2_Cl_2_-MeOH and leaves of* Strychnos usambarensis* (50% MeOH in CH_2_Cl_2_) with IC_50_ values of 9.04, 9.43, and 11.09 *μ*g/mL. Similar results were obtained for the extract from the leaves of* Vepris soyauxii* (IC_50_ 9.28 *μ*g/mL), the whole plant of* Gladiolus quartinianus* (IC_50_ 10.57 *μ*g/mL) [42], and the bark of* Nauclea pobeguinii* (IC_50_ 14.62 *μ*g/mL), [43].

The results obtained in this work against CCRF/CEM cell lines (IC_50_ 18.11 *μ*g/mL) are consistent with those of the leaves of* Anonidium mannii* (IC_50_ 17.32 *μ*g/mL), [43].

According to the National Cancer Institute (United States) plant screening program, a crude extract is generally considered to have* in vitro* cytotoxic activity if the IC_50_ is <20 *μ*g/mL. In turn, the criteria of the ATCC have established a concentration of 30 *μ*g/mL as the upper IC_50_ limit [41]. On the basis of this threshold, the intractum tested in our study can be considered as a promising cytotoxic agent towards HL-60, HL-60/MX1, and CCRF/CEM cell lines (IC_50_ < 20 *μ*g/mL).

### 3.6. Antioxidant Activity and the Structure-Activity Relationship

**Figure 4:**
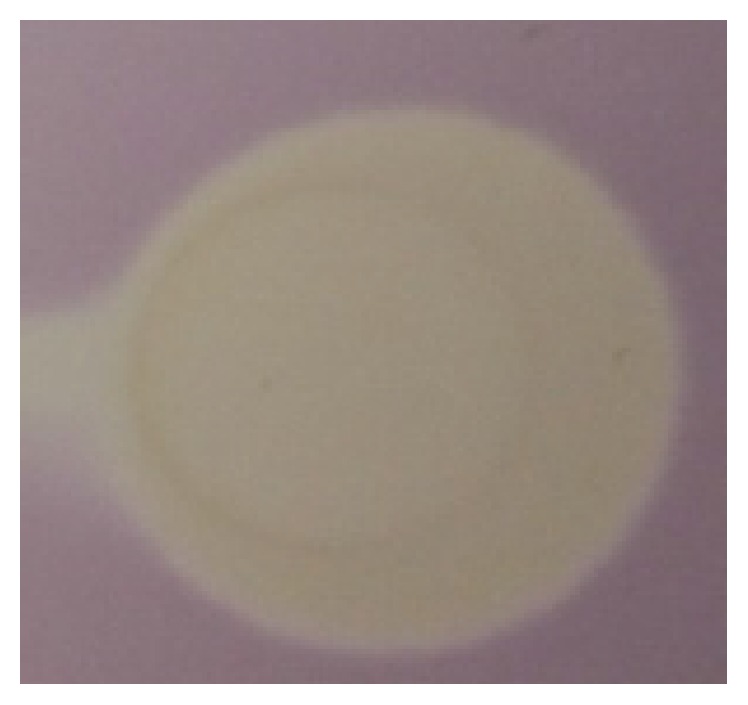
A “dot-blot” TLC-DPPH^*∗*^ test performed on silica gel plate, 1 min after immersion in DPPH^*∗*^ methanolic solution.

**Figure 5:**
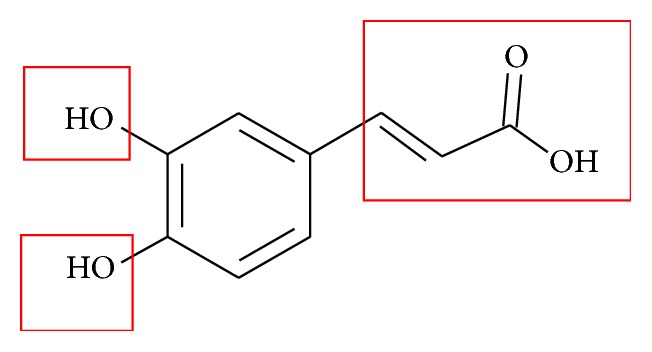
Structure of* trans*-caffeic acid (groups marked in red participate in an interaction of free radical with caffeic acid).

The human diet provides antioxidants from plant sources in order to protect the body from the radical damage at the biochemical and molecular levels. Antioxidant capacities of the extracts were expressed in terms of EC_50_ value. It was found that the intractum was able to effectively reduce free radicals, with the EC_50_ value of 52 *μ*g/mL and ascorbic acid and*α*-tocopherol 40 and 5.0 *μ*g/mL, respectively. Free radical scavenging activity has been confirmed by means of TLC-DPPH^*∗*^ dot-blot test using silica gel as the stationary phase (Figure 4). Regions of the TLC plate which contain DPPH^*∗*^ inhibitors show up as yellow spots against a purple background. We observed the plate after 1, 5, 10, and 30 min from the time of immersion of the plate in 0.2 mmol DPPH^*∗*^ solution. After 1 min, the intractum showed area of activity at concentration 10 *μ*g/spot. It is noteworthy that, in the spectrophotometric assay, the intractum showed strong antiradical properties, and this was confirmed in the autography test. It suggests the presence of the compounds which are able to donate hydrogen or electron and may be considered as antioxidants. The antioxidative molecular mechanism can be based on the chemical structure of phenolic acids, especially* trans*-caffeic, that possess in their structure the two OH groups, one in the -*para* position and known as a strong antioxidant (Figure 5) [44]. The propenyl chain can also improve the efficiency due to the presence of the double C=C bond. In our previous study we reported that the leaves and fruits of* Eleutherococcus* species have a high antioxidant capability [8, 9, 19].

## 4. Conclusion

We assume that this study provides the better understanding of pharmacological activity of* E. senticosus* and confirms ethnopharmacological reports on rightness of using that species in traditional medicine. The administration of the intractum could help to improve the body's shape. A special attention should be paid to an incorporation of the species in commercial or domestic cultivations, which would allow for their valuable exploitation. Further studies are needed to focus on exploring the mechanism of action of constituents in* in vivo* model.

## Supplementary Material

Supplementary Material reports LC-ESI-MS/MS analytical results of phenolic acids and analytical parameters of LC-ESI-MS/MS quantitative method; data for calibration curves, limit of detection (LOD) and limit of quantification (LOQ) values for each analyzed compound.

## Figures and Tables

**Table 1:** TPC and TFC in intractum from the fruits of *E. senticosus* (mg GAE/mL, QEs/mL, HEs/mL, mg/per serving, and mg/g dry extract). One serving = 25 mL for intractum.

TPC	Flavonoid content		TFC
	FeCl_3_	DNPH	
mg/mL mg/per serving mg/g exc. ±SD	mg/mL mg/per serving mg/g exc. ±SD	mg/mL mg/per serving mg/g exc. ±SD	mg/mL mg/per serving mg/g exc. ±SD
2.6 ± 0.11	0.37 ± 0.12	1.47 ± 0.12	1.84 ± 0.12
65 ± 0.12	9.25 ± 0.12	36.75 ± 0.12	46 ± 0.12
130 ± 0.55	18.5 ± 0.23	73.5 ± 0.34	92 ± 0.36

The data are represented as the mean (±SD) of three independent measurements.

**Table 2:** Concentrations of phenolic acids in the raw intractum and after acidic hydrolysis (mg/g DE).

Compound	Raw intractum	Ethyl acetate layer	Dichloromethane layer
Protocatechuic	0.45 ± 2.1	0.10 ± 4.2	0.1 ± 2.1
Gentisic	—	0.004 ± 0.02	0.01 ± 0.7
4-OH-benzoic	2 ± 1.4	7.0 ± 0.3	7.3 ± 0.7
Vanillic	4.2 ± 0.2	21.1 ± 6.3	16.0 ± 4.7
*trans*-Caffeic	41.2 ± 2.8	—	—
Syringic	—	0.8 ± 0.4	—
*trans*-*p*-Coumaric	2.5 ± 2.1	—	—
*trans*-Ferulic	3.6 ± 0.9	—	—

**Table 3:** Mineral compositions of fruits [mg/kg of dry weight].

Ca	Mg	K	Na	Fe	Cu	Zn	Mn
1750	1300	21000	218	32.7	4.35	23.5	54.3

**Table 4:** Inhibition of Hyal (%) for a different activity unit of enzyme (mIU/mg protein).

Sample	mIU/mg protein				
	30	40	53	90	109
Intractum	60 ± 2.3	31.4 ± 1.3	5.1 ± 0.3	2.1 ± 0.4	n.s.
Aescin	50 ± 1.8	29.3 ± 0.9	13.5 ± 1.5	n.s.	n.s.
Naringenin	38.9 ± 2.9	15.4 ± 2.1	n.s.	n.s.	n.s.

n.s.: not showed.

**Table 5:** IC_50_ (*μ*g/mL) of the intractum and podophyllotoxin.

Cell line	CEM/C1	CCRF/CEM	HL-60	HL-60/MX1	HL-60/MX2
Intractum	30.4	18.1	10.4	15.7	50
Podophyllotoxin	0.0286	0.0064	0.0085	0.0078	0.0106

## Abbreviation

TPC: Total phenolics content
TFC: Total flavonoids content
DPPH^*∗*^: 2,2-Diphenyl-1-picrylhydrazyl
DNPH: 2,4-Dinitrophenylhydrazine
FeCl_3_: Aluminium chloride
GA: Gallic acid
HE: Hesperetin equivalent
QE: Quercetin equivalent
TLC-DB-DPPH dot-blot test: TLC-Direct Bioautography dot-blot test
AAS: Atomic absorption spectroscopy
Hyal: Hyaluronidase
HA: Hyaluronic acid
HL-60: Human Caucasian promyelocytic leukemia from American Type Culture Collection (ATCC CCL-240™)
HL-60/MX1: Human Caucasian acute promyelocytic leukemia from American Type Culture Collection (ATCC CRL-2258™)
HL60/MX2: Human Caucasian acute promyelocytic leukemia from American Type Culture Collection (ATCC CRL-2257™)
CEM/C1: Human Caucasian acute lymphoblastic leukemia from American Type Culture Collection (ATCC CRL-2265™)
CCRF/CEM: Human Caucasian acute lymphoblastic leukemia from American Type Culture Collection (ATCC CCL-119™).
