# Supplementary material for: Phytochemical Content and Pharma-Nutrition Study on Eleutherococcus senticosus Fruits Intractum
Source: Oxid Med Cell Longev. 2016 Oct 24;2016:9270691. doi: 10.1155/2016/9270691 (PMC5098108; doi:10.1155/2016/9270691)
Supplement: Supplementary file 1 — Supplementary Material reports LC-ESI-MS/MS analytical results of phenolic acids and analytical parameters of LC-ESI-MS/MS quantitative method; data for calibration curves, limit of detection (LOD) and limit of quantification (LOQ) values for each analyzed compound. [file 9270691.f1.pdf]

TABLES

Table S1: LC-ESI-MS/MS analytical results of phenolic acids. Compounds confirmed by comparison with authentic standards.

Compound	Peak no.	T_R (min)	$[M-H]^-$	Fragment ions	Collision energy (eV)
**Gallic acid**	1	1.10	168.7	124.9	- 14
				78.9	- 36
**Protocatechuic acid**	2	2.25	152.9	107.8	- 38
				80.9	- 26
**Gentisic acid**	3	4.15	152.8	107.9	- 36
				81	- 30
**4-OH-benzoic acid**	4	4.30	136.8	92.9	- 18
**3-OH-benzoic acid**	5	5.40	136.8	92.9	- 18
				75	- 48
**Vanillic acid**	6	5.65	166.8	107.9	- 18
				123	- 12
***trans*-Caffeic acid**	7	5.90	178.7	134.9	- 16
				88.9	- 46
***cis*-Caffeic acid**	8	6.20	178.7	134.9	- 16
				88.9	- 46
**Syringic acid**	9	6.80	196.9	181.9	- 12
				122.8	- 24
***trans-p*-Coumaric acid**	10	7.70	162.7	119	- 14
				93	- 44
***cis-p*-Coumaric acid**	11	7.81	162.7	119	- 14
				93	- 44
***trans*-Ferulic acid**	12	8.25	192.8	177.9	- 12
				133.9	- 16
**Veratric acid**	13	8.30	180.7	136.9	- 12
				121.9	- 18
**Salicylic acid**	14	8.38	136.8	93	- 16
				75	- 48
**3-OH-cinnamic acid**	15	8.40	162.8	119	- 14
				91	- 36
***cis*-Ferulic acid**	16	8.40	192.8	177.9	- 12
				133.9	- 16
***trans*-Sinapic acid**	17	8.42	222.8	148.9	- 20
				121	- 36
***cis*-Sinapic acid**	18	8.58	222.8	148.9	- 20
				121	- 36

Table S2: Analytical parameters of LC-MS/MS quantitative method; data for calibration curves, limit of detection (LOD) and limit of quantification (LOQ) values for each analyzed compound.

Compound	LOD [ng mL^{1}]	LOQ [ng mL^{-1}]	Calibration curve	r^2	Linearity range [ng mL^{-1}]
Gallic acid	25	50	y = 218x -201	0.9995	50-5000
Protocatechuic acid	10	25	y = 86.2x + 1260	0.9999	50-12500
Gentisic acid	5	15	y = 851x + 6880	0.9994	15-5000
4-OH-benzoic acid	20	50	y = 1480x + 6490	0.9998	50-2000
3-OH-benzoic acid	20	40	y = 415x + 11400	0.9999	50-2000
Vanilic acid	25	50	y = 59.4x + 1740	0.9994	50-5000
trans-Caffeic acid	10	25	y = 1100x + 4040	0.9994	25-2500
cis-Caffeic acid	10	25	y = 1100x + 4040	0.9994	25-2500
Syringic acid	25	50	y = 75.4x + 4230	0.9996	50-10000
trans- *p*-Coumaric acid	10	25	y = 888x + 403	0.9999	25-2500
cis-*p*-Coumaric acid	10	25	y = 888x + 403	0.9999	25-2500
trans-Ferulic acid	10	25	y = 411x - 1840	0.9994	40-2000
Veratric acid	100	200	y = 102x + 2470	0.9996	200-5000
Salicylic acid	10	30	y = 2060x + 14300	0.9999	30-1000
3-OH-cinnamic acid	10	25	y = 569x + 4280	0.9997	50-2500
cis-Ferulic acid	10	25	y = 411x - 1840	0.9994	40-2000
trans-Sinapic acid	8	20	y = 319x - 33.3	0.9997	20-2500
cis-Sinapic acid	8	20	y = 319x - 33.3	0.9997	20-2500
